# “Biggs bang” – when alignment truly ignites

**DOI:** 10.3205/zma001794

**Published:** 2025-11-17

**Authors:** Thomas Rotthoff

**Affiliations:** 1 University of Augsburg, Faculty of Medicine, Medical Didactics and Educational Research (DEMEDA), Augsburg, Germany

## Editorial

In medical curricula development, *constructive alignment* is regarded as central to effective teaching, learning, and assessment. In practice, the focus is often primarily on alignment—that is, the substantive and methodological coordination of learning objectives, teaching formats, and assessments. The term *constructive* is frequently interpreted more implicitly and descriptively in the sense of “constructing”: The curriculum is designed so that learning objectives, teaching formats, and assessments are coherently aligned. However, constructive alignment – a concept attributed primarily to John Biggs [1] – is grounded in constructivist learning theory, in which learning is seen as the progressive process of structuring content, with learners actively and independently building knowledge and understanding through meaningful learning activities. The key to learning processes is thus not merely the transmission of content but the creation of learning situations in which relationships between knowledge elements and their interconnections can be developed [1]. The depth and complexity of the knowledge or understanding a learner demonstrates beyond defining what they should know or be able to do is therefore relevant [2]. 

The semantic shift of constructive from “constructivist” to “constructing” is often attributed – particularly in Europe – to the Bologna Process and the development of a common European higher education framework [3]. In this context, attention is increasingly shifting toward learning outcomes and less – contrary to Biggs’s original intent – toward the processes of teaching and learning. Although medical education in Germany is not formally part of the Bologna Process, a similar outcome orientation has been adopted through the* National Competence-Based Learning Objectives Catalogue for Medicine *(NKLM). Outcomes in medical education are now typically linked to appropriate teaching and assessment formats in line with the principle of alignment. Nevertheless, what actually happens within these formats in terms of teaching and learning processes is receiving less attention. Assigning a teaching format to a learning objective does not automatically guarantee that active learning will take place during its implementation. An increasing tendency is to assume that competence orientation in medical education is achieved simply by repeatedly invoking the term and defining the associated competencies and outcomes – without these necessarily being fully reflected in the actual design of teaching sessions. What truly matters is what happens in the teaching session itself and how learning is stimulated. Active learning is characterized by attributes such as analysis and synthesis, application and critical reflection, correcting misconceptions, questioning established interpretations, and motivational aspects (e.g., autonomy, competence experience). Competence acquisition in medical education is a complex process: It occurs across multiple levels and through various pathways [4] and is particularly enhanced by teaching formats that promote active, reflective, and context-bound learning. Whether deeper cognitive processes are genuinely triggered depends largely on the teacher’s didactic skills [5]. Effective teachers can present core concepts and content clearly and vividly, design suitable tasks and problems, activate and guide learning processes, monitor learning progress, provide feedback, and thus influence student learning outcomes [5]. This requires not only subject-matter expertise but also didactic competence. In large student cohorts, the small-group formats best suited for competence development require numerous groups. The corresponding teaching responsibilities often run in parallel to clinical duties and are distributed among many physicians. These clinical teachers frequently have no formal training in education, and junior physicians at the beginning of their specialty training lack sufficient clinical expertise. With assessments looming, attempts are often made to secure teaching quality through maximum standardization, for example, by having different teachers use the same presentation for different student groups, to ensure that all students receive identical content to create efficiency and to give teachers a sense of security. However, under such conditions, cognitively demanding and adaptive teaching situations – which require didactic understanding and practice – are often missed. Creative teaching design and authentic active learning are thus lost, and teaching risks becoming a mere exercise in delivering standardized instruction [6]. 

Given the current strong focus on outcomes, the question arises as to whether the potential of constructive alignment in medical education is being fully exploited as a consistently competence-oriented approach that involves active engagement, problem-solving, discussion, and application. One thing is clear: In recent years, medical education in Germany has made tremendous strides. The qualification of teaching staff has gained significant importance and yielded visible successes. At many institutions, formal training in medical education is now an expected and binding component of the habilitation process. To sustainably and comprehensively improve teaching quality, however, more teachers must be involved so that everyone, in their respective roles, has the necessary knowledge and didactic competence [7]. From a faculty perspective, this means training educators so that they can create active learning environments and contribute to shaping the curriculum [3], [8]. Given increasingly limited resources, it is crucial to organize teaching funding in such a way that it strongly incentivizes the didactic development of as many teachers as possible. At the same time: personnel-intensive teaching formats that are not utilized effectively constitute an inefficient allocation of resources in medical education from an economic perspective. Constructive alignment will only truly ignite into a “Biggs bang” when educators fully embrace and competently enact their roles – to the benefit of students and their own professional satisfaction.

The relevance of developing teachers' skills is highlighted in several articles in this issue. Gehrke-Beck et al. [9] show that nationally structured accreditation and qualification programs are needed to ensure the cross-location qualification of general medical teaching practices. Preti et al. [10][ report that “expertise role reversal” – in which learners contribute their knowledge and teachers temporarily take on the role of learners – can strengthen pedagogical competence and promote an open, learning-oriented teaching culture. Hennel et al. [11] recommend that interdisciplinary aspects such as professional identity formation and career planning be given greater consideration in feedback and examination situations. Multisource feedback (MSF) can provide individual feedback from different perspectives; however, targeted further training of teachers is also necessary to ensure the quality of this feedback. These studies emphasize that the continuous professionalization of teachers is an important prerequisite for high-quality medical teaching.

## Author’s ORCID

Thomas Rotthoff: [0000-0002-5171-5941]

## Use of AI

The title of the editorial was developed using prompting in ChatGPT 5.0. 

## Competing interests

The author declares that he has no competing interests.
